# Supplementary material for: Morphological characterization and DNA barcoding of Ruellia sp. in Saudi Arabia
Source: PLoS One. 2025 Feb 19;20(2):e0315827. doi: 10.1371/journal.pone.0315827 (PMC11838864; doi:10.1371/journal.pone.0315827)
Supplement: S1 Table — (DOCX) [file pone.0315827.s001.docx]

**S1 Table. Morphological characteristics of plant species recorded during the study**

| Taxa | Height Of the Plant M | The Plant Color | Stem | | | | | leaf shape | pedicel Height(cm | Calyx shape | Corolla color | Fruit | | |
| --- | --- | --- | --- | --- | --- | --- | --- | --- | --- | --- | --- | --- | --- | --- |
|  |  |  | Internode shorter | Glabrous | Pubescent | Cylindrical | quadrangular |  |  |  |  | Petiole | Color before ripe | Shape |
| ***R.sp.Abha*** | 23.3 | green | *+* | + | *-* | *-* | + | Lanceolate | 2 | lanceolate | Violat | + | Green | Elliptic, narrowing from below |
| ***R.sp.24651*** | 3.7 | green | *-* | *-* | + | *+* | + | Ovate | 1 | Elliptic | Violat&wh-ite | - | Green | oval with a narrow end |
| ***R.sp.24652*** | 11.5 | green | *-* | *+* | - | *-* | + | Linear | 1 | Linear | Rose | + | Green & Red | Cylindrical narrowing from above and below |
| ***R.simplex -R.malacosperma*** | 8.3 | Brown-purple | *-* | + | *-* | *-* | + | Lanceolate | 1.2 | Linear | Violat | + | Green & Red | Oblong |
| ***R.patula*** | 5.5 | green | *-* | - | *+* | *-* | + | Cordate | 1 | Linear | Violat&wh-ite | + | Green | Orbicular to ovule |
| ***R.sp.24650-rose*** | 10.9 | green | *+* | + | *-* | *-* | + | Linear | 0.8 | Elliptic | Pale rose | + | Green | Cylindrical, narrowing at the top |
| ***R.sp.24650-violat*** | 12.8 | Brown-purple | *-* | + | *-* | *-* | + | Linear | 0.8 | Elliptic | Violat | + | Green | Fusiform |
| ***R.sp.24650-white*** | 15.7 | green | *+* | + | *-* | *-* | + | Linear | 2cm | Elliptic |  | + | Green | elliptic narrowing from below |
