# Supplementary material for: Morphological characterization and DNA barcoding of Ruellia sp. in Saudi Arabia
Source: PLoS One. 2025 Feb 19;20(2):e0315827. doi: 10.1371/journal.pone.0315827 (PMC11838864; doi:10.1371/journal.pone.0315827)
Supplement: Attachment — Submitted filename: PONE-D-24-30210_reviewer_10.09.2024.pdf [file pone.0315827.s003.pdf]

PLOS ONE

Morphological characterization and (DNA) Barcoding identification of Ruellia spp. (Acanthaceae) in Saudi Arabia

--Manuscript Draft--

**Manuscript Number:**	PONE-D-24-30210
**Article Type:**	Research Article
**Full Title:**	Morphological characterization and (DNA) Barcoding identification of Ruellia spp. (Acanthaceae) in Saudi Arabia
**Short Title:**	Morphological characterization and DNA Barcoding identification of Ruellia spp. (Acanthaceae) in Saudi Arabia
**Corresponding Author:**	MANAR D ALSHEHRI, Assistant Professor<br>King Khalid University College of Science<br>ABHA, SAUDI ARABIA
**Keywords:**	morphology; phylogenetic; Analysis; DNA barcoding; Ruellia; genotype
**Abstract:**	This study is recognized as the first taxonomic investigation of Ruellia species within the Kingdom of Saudi Arabia. The primary objective is to identify the various species within the genus Ruellia using both morphological characteristics and DNA barcoding methods. For this purpose, plant samples were meticulously collected from eight distinct natural habitats across the region. To ensure accurate identification and characterization, four molecular barcoding markers were employed: Psbk-psbi, trnH-psbA, rbcL, and AtpF-AtpH. Eight Ruellia species were identified from different regions: Abha, Aseer (24651), Jazan (24652), Malacosperma, Patula, Taif (24650 rose), Taif (24650 violet), and Taif (24650 white). The species were confirmed using specimens from the King Saud University herbarium. Notably, the samples collected from Taif, which had flowers of different colors, were determined to represent a single species with different genotypes. The use of four DNA barcode markers (Psbk-psbi, trnH-psbA, rbcL, and AtpF-AtpH) facilitated the identification of five distinct species: R. tweediana, R. sp. SH2010, R. carolinensis, R. simplex, and R. patula. These findings confirmed the dominant Ruellia species in Saudi Arabia and demonstrated the reliability of DNA barcode markers for species identification. Further assessment of these species' adaptability, molecular genetics, and functional genomics is necessary for their commercial utilization in the region. These species are recorded for the first time in Saudi Arabia and represent the first record.
**Order of Authors:**	Jawaher S. Almuteri, Phd Student
	Mona S. Al wahaibi, Professor
	Abd El-Zaher M. A. Mustafa
	Manal A. Alshaqhaa, Assistant Professor
	Muhammed Afzal
	MANAR D ALSHEHRI, Assistant Professor
**Additional Information:**	
**Question**	**Response**
**Financial Disclosure**<br><br>Enter a financial disclosure statement that describes the sources of funding for the work included in this submission. Review the submission guidelines for detailed requirements. View published research articles from *PLOS ONE* for specific examples.	Yes

Powered by Editorial Manager® and ProduXion Manager® from Aries Systems Corporation

This statement is required for submission and **will appear in the published article** if the submission is accepted. Please make sure it is accurate.<br><br>**Funded studies**<br>Enter a statement with the following details:<br>• Initials of the authors who received each award<br>• Grant numbers awarded to each author<br>• The full name of each funder<br>• URL of each funder website<br>• Did the sponsors or funders play any role in the study design, data collection and analysis, decision to publish, or preparation of the manuscript?<br><br>Did you receive funding for this work?	
Please add funding details.<br>as follow-up to "**Financial Disclosure**<br><br>Enter a financial disclosure statement that describes the sources of funding for the work included in this submission. Review the submission guidelines for detailed requirements. View published research articles from *PLOS ONE* for specific examples.<br><br>This statement is required for submission and **will appear in the published article** if the submission is accepted. Please make sure it is accurate.<br><br>**Funded studies**<br>Enter a statement with the following details:<br>• Initials of the authors who received each award<br>• Grant numbers awarded to each author<br>• The full name of each funder<br>• URL of each funder website<br>• Did the sponsors or funders play any role in the study design, data collection and analysis, decision to publish, or preparation of the manuscript?	Deanship of Research and Graduate Studies at King Khalid University submitted the funding for this work through small group research under grant number RGP1/200/45.

Powered by Editorial Manager® and ProduXion Manager® from Aries Systems Corporation

Did you receive funding for this work?"	
Please select the country of your main research funder (please select carefully as in some cases this is used in fee calculation).<br>as follow-up to "**Financial Disclosure**<br><br>Enter a financial disclosure statement that describes the sources of funding for the work included in this submission. Review the submission guidelines for detailed requirements. View published research articles from *PLOS ONE* for specific examples.<br><br>This statement is required for submission and **will appear in the published article** if the submission is accepted. Please make sure it is accurate.<br><br>**Funded studies**<br>Enter a statement with the following details:<br>• Initials of the authors who received each award<br>• Grant numbers awarded to each author<br>• The full name of each funder<br>• URL of each funder website<br>• Did the sponsors or funders play any role in the study design, data collection and analysis, decision to publish, or preparation of the manuscript?<br><br>Did you receive funding for this work?"	SAUDI ARABIA - SA
**Competing Interests**<br><br>Use the instructions below to enter a competing interest statement for this submission. On behalf of all authors, disclose any competing interests that could be perceived to bias this work—acknowledging all financial supportand any other relevant financial or non-financial competing interests.<br><br>This statement is required for submission	The authors have declared that no competing interests exist.

and **will appear in the published article** if the submission is accepted. Please make sure it is accurate and that any funding sources listed in your Funding Information later in the submission form are also declared in your Financial Disclosure statement.<br><br>View published research articles from *PLOS ONE* for specific examples.<br><br>**NO authors have competing interests**<br><br>Enter: *The authors have declared that no competing interests exist.*<br><br>**Authors with competing interests**<br><br>Enter competing interest details beginning with this statement:<br><br>*I have read the journal's policy and the authors of this manuscript have the following competing interests: [insert competing interests here]*<br><br>* typeset	
**Ethics Statement**<br><br>Enter an ethics statement for this submission. This statement is required if the study involved:<br><br>• Human participants<br>• Human specimens or tissue<br>• Vertebrate animals or cephalopods<br>• Vertebrate embryos or tissues<br>• Field research<br><br>Write "N/A" if the submission does not require an ethics statement.<br><br>General guidance is provided below. Consult the submission guidelines for detailed instructions. **Make sure that all information entered here is included in the Methods section of the manuscript.**	N/A

Powered by Editorial Manager® and ProduXion Manager® from Aries Systems Corporation

**Format for specific study types**<br><br>**Human Subject Research (involving human participants and/or tissue)**<br>• Give the name of the institutional review board or ethics committee that approved the study<br>• Include the approval number and/or a statement indicating approval of this research<br>• Indicate the form of consent obtained (written/oral) or the reason that consent was not obtained (e.g. the data were analyzed anonymously)<br><br>**Animal Research (involving vertebrate animals, embryos or tissues)**<br>• Provide the name of the Institutional Animal Care and Use Committee (IACUC) or other relevant ethics board that reviewed the study protocol, and indicate whether they approved this research or granted a formal waiver of ethical approval<br>• Include an approval number if one was obtained<br>• If the study involved *non-human primates*, add *additional details* about animal welfare and steps taken to ameliorate suffering<br>• If anesthesia, euthanasia, or any kind of animal sacrifice is part of the study, include briefly which substances and/or methods were applied<br><br>**Field Research**<br><br>Include the following details if this study involves the collection of plant, animal, or other materials from a natural setting:<br>• Field permit number<br>• Name of the institution or relevant body that granted permission	
**Data Availability**<br><br>Authors are required to make all data underlying the findings described fully available, without restriction, and from the time of publication. PLOS allows rare exceptions to address legal and ethical concerns. See the PLOS Data Policy and FAQ for detailed information.	Yes - all data are fully available without restriction

A Data Availability Statement describing where the data can be found is required at submission. Your answers to this question constitute the Data Availability Statement and **will be published in the article**, if accepted.<br><br>**Important:** Stating 'data available on request from the author' is not sufficient. If your data are only available upon request, select 'No' for the first question and explain your exceptional situation in the text box.<br><br>Do the authors confirm that all data underlying the findings described in their manuscript are fully available without restriction?	
**Describe where the data may be found in full sentences. If you are copying our sample text, replace any instances of XXX with the appropriate details.**<br><br>• If the data are **held or will be held in a public repository**, include URLs, accession numbers or DOIs. If this information will only be available after acceptance, indicate this by ticking the box below. For example: *All XXX files are available from the XXX database (accession number(s) XXX, XXX.*).<br>• If the data are all contained **within the manuscript and/or Supporting Information files**, enter the following: *All relevant data are within the manuscript and its Supporting Information files.*<br>• If neither of these applies but you are able to provide **details of access elsewhere**, with or without limitations, please do so. For example:<br><br>*Data cannot be shared publicly because of [XXX]. Data are available from the XXX Institutional Data Access / Ethics Committee (contact via XXX) for researchers who meet the criteria for access to confidential data.*<br><br>*The data underlying the results presented in the study are available from (include the name of the third party*	All relevant data are within the manuscript and its Supporting Information files.

and contact information or URL).<br>• This text is appropriate if the data are owned by a third party and authors do not have permission to share the data.<br><br>* typeset	
Additional data availability information:	Tick here if your circumstances are not covered by the questions above and you need the journal's help to make your data available.

Powered by Editorial Manager® and ProduXion Manager® from Aries Systems Corporation

Manuscript

Click here to access/download;Manuscript;Ruellia spp. (Acanthaceae) in Saudi Arabia.docx

Morphological characterization and (DNA) Barcoding identification of *Ruellia spp*. (Acanthaceae) in Saudi Arabia

Jawaher S. Almuteri[1] Mona S. Al wahaibi[1], Abd El-Zaher M. A. Mustafa[1] Manal A. Alshaqhaa [2] Muhammed Afzal[3]Manar D.Alshehri[2]

[1]Botany and Microbiology Department, College of Science, King Saud University, Riyadh, Saudi Arabia
[2] Biology Department, College of Science, King Khalid University, Abha 61413, Saudi Arabia
3 College of Food and Agricultural Sciences, King Saud University, Saudi Arabia

Abstract

This study is recognized as the first taxonomic investigation of *Ruellia* species within the Kingdom of Saudi Arabia. The primary objective is to identify the various species within the genus *Ruellia* using both morphological characteristics and DNA barcoding methods. For this purpose, plant samples were meticulously collected from eight distinct natural habitats across the region. To ensure accurate identification and characterization, four molecular barcoding markers were employed: Psbk-psbi, trnH-psbA, rbcL, and AtpF-AtpH. Eight *Ruellia* species were identified from different regions: Abha, Aseer (24651), Jazan (24652), Malacosperma, Patula, Taif (24650 rose), Taif (24650 violet), and Taif (24650 white). The species were confirmed using specimens from the King Saud University herbarium. Notably, the samples collected from Taif, which had flowers of different colors, were determined to represent a single species with different genotypes. The use of four DNA barcode markers (Psbk-psbi, trnH-psbA, rbcL, and AtpF-AtpH) facilitated the identification of five distinct species: *R. tweediana*, *R. sp. SH2010, R. carolinensis*, *R. simplex,* and *R. patula*. These findings confirmed the dominant *Ruellia* species in Saudi Arabia and demonstrated the reliability of DNA barcode markers for species identification. Further assessment of these species' adaptability, molecular genetics, and functional genomics is necessary for their commercial utilization in the region. These species are recorded for the first time in Saudi Arabia and represent thefirst record.

Kay words: morphology- phylogenetic- analysis- DNA barcoding- *Ruellia*-genotype

Introduction

Commented [MS1]: Please revise the title as 'Morphological characterization and DNA barcoding of *Ruellia* sp. in Saudi Arabia'

Commented [MS2]: Please write the full name of the authors

Commented [MS3]: Please re-write the abstract highlighting the importance of the crop, the need of genetic discrimination study, methodology, key findings, and future thrust. You may claim the first report at the end, not at the beginning.

Commented [MS4]: Please delete the sentense

Commented [MS5]: Please write the gene name in italics throughout the text

Commented [MS6]: The introduction is poorly written. Please provide a brief introduction of the plant and its importance. Research gap and the novelty of the study to bridge the research gap. Introduce the barcode genes and justify why you have chosen the primers. Please justify not using other barcode primers, chloroplast-plastid, and nuclear region. Please elaborate on the importance of DNA barcoding-aided specified delamination and its future implications. Please make more reviews of literature in the same or different species.

The family Acanthaceae is distributed mainly in four places in the world, Indonesia, Malaysia, Africa, and Central America. This family is also represented in the Kingdom of Saudi Arabia with 24 genera and 35 species (Al farhan et al, 2005). The genus *Ruellia* L. belongs to this family, and its plants are herbs or shrubs (Tripp, 2007). This genus was first detected in the tropical and subtropical regions. It includes about 350 species globally (Tripp et al., 2013). *Ruellia* holds medicinal significance due to the presence of benzoxazinoids in *R. tuberose* (Samy et al., 2013). These compounds are utilisedutilized in the treatment of stomach cancer and leukemia leukaemia (Graham et al., 2000; Reddy et al., 1991), as well as for managing skin diseases and abscesses (Selvam, 2008), ocular ailments (Kirtikar and Basu, 1935), and bronchitis (Chiu and Chang, 1995). Plant-specific barcodes enhance our ability to distinguish closely related plants at species and genotypes level (Li, 2015). The genetic barcoding of DNA is very important for researchers in the field of taxonomy (Gregory, 2005). DNA barcoding will help to identify known species, discover thousands of species yet to be named, and manage the vast biodiversity on Earth (Ali et al., 2014). The most common DNA gene barcoding used for plants is two strands of the large subunit chloroplast gene RuBisCo (rbcL) and the maturase K matK genes (Heber et al., 2003). Khan et al., (2019) studied some selected genera of Acanthaceae (2019) studied some selected genera of Acanthaceae, including the genus *Ruellia* based on large plastid genes, of which the DNA (cp-DNA) genes rbcL, matK and the spacer trnH-psbA genes. They found that rbcL is more efficient, while a combination of matK and trnH-psbA can be used to identify and study the phylogenetics of plants. No previous taxonomic studies on the plants of the genus *Ruellia* in KSA were found. This study employed DNA barcoding, in addition to phenotypic description, on several plant samples from the genus *Ruellia*. DNA barcoding was chosen for its capacity for accurate plant identification (Li, 2015) owning to its high quality (Dong et al., 2013).

Materials and Methods:

Plant Samples:

The samples were gathered from their natural environment, and herbarium specimens were subsequently prepared and registered at the herbarium of King Saud University. Detailed information about the collection locations and herbarium numbers of each plant sample presented in Table 1.

Commented [MS7]: Mention the country name for all the places

Table 1. List of Plant samples of the genus *Ruellia* studied and collection sites in Saudi Arabia
* Herbarium number

Taxa	Collection place	Collection date	GPS coordinates
1. *Ruellia sp.Abha*	Abha	December, 2021	18°16'37.6"N 42°43'23.5"E
2. *Ruellia sp.24651* *	Aseer	December, 2021	19°07'44.5"N 41°55'42.2"E
3. *Ruellia sp.24652* *	Jazan	September, 2021	16°59'30.7"N 42°42'59.0"E
4. *Ruellia malacosperma*	Riyadh, Jazan, Madinah	September, 2021	24°43'07.9"N 46°37'24.4"E
5. *Ruellia patula*	Jazan	September, 2021	16°54'50.1"N 42°33'20.4"E
6. *Ruellia .sp.24650rose* *	Taif	December, 2021	21°30'34.8"N 40°29'17.2"E
7. *Ruellia.sp.24650. Violet* *	Taif	December, 2021	21°30'34.8"N 40°29'17.2"E
8. *Ruellia*.sp.24650 White	Taif	December, 2021	21°30'34.8"N 40°29'17.2"E

The plant samples used in the current study were collected from eight natural habitats (Figure 1). The shoot system, including the stem and leaves, was examined for the blade shape, edge, base, and tip. Additionally, the inflorescence, flower density on the axis, calyx, corolla, stamens, and pistils were analyzed.

DNA extraction:

Genomic DNA was extracted from specimens by crushing the tissue in liquid nitrogen, applying modified CTAB method (Doyle & Doyle, 1987). The acquired pellet was cleaned with 70% ethanol and permitted to dry at room temperature. DNA pellet suspended in T.E. buffer was quantified by using a UV Spectrophotometer with A260/280 ranges from 1.62-1.78.

Primers: PCR amplification trnH-psbA gene primers ,psbK-psbI F: TTAGCATTTGTTTGGCAAG R:AAAGTTTGAGAGTAAGCAT, trnH-psbA F: GTTATGCACGAACGTAATGCTC R: CGCGCGTGGTGGATTCACAATCC atpF-

Commented [MS8]: Why the sample 1,4,5, don't have herbarium number

Commented [MS9]: Please elaborate the sample collection and processing.

Commented [MS10]: What modification you have done. Please explain

Commented [MS11]: Please mention the full form

Commented [MS12]: Please provide the details in a tabular for with Tm

Commented [MS13R12]: Please mention the Company/place of primer synthesis

atpH F: ACTCGCACACACTCCCTTTCC R: GCTTTTATGGAAGCTTTAACAAT (Wang et al., 2010) , rbcL F: GTAAAATCAAGTCCACCACG R: ATGTCACCACAAACAGAGACTAAAGC (Suzuki et al., 2014). ~~were applied.~~

PCR Amplification and Sequencing:

A PCR reaction was carried out in 0.2 ml PCR tubes by using 25µl total volume to amplify the desired markers. The valuable loci were amplified by using a robust PCR kit (Applied Bio systems). For the *matK*, the cycling conditions were 95°C for 10 minutes, 30 cycles at 95°C for the 30s, 55°C for 45s, 72°C for 1min; and the final elongation at 72°C for 4min. The *rbcL* primers were cycled at 95°C for 10 min, 30 cycles at 58°C for the 30s, 72°C for 1 min, and finally for 4 min, at 72°C for final elongation. PCR products were separated and ~~were~~ visualized on 1% agarose gel and purified through a DNA purification kit (Thermo Scientific). The purified PCR samples were sent to Macrogen in South Korea (https://www.macrogen.com/en/main/index.php, accessed on 15~~,~~ June 2022) for sequencing using an ABI3730 automated sequencer with the same markers as in the PCR reactions. Both DNA strands were sequenced and checked for ambiguous nucleotides. The first 30 base pairs of each read were removed, the length of the amplicon products was measured, and multiple DNA sequence alignments were generated using ClustalW alignment software (Thompson et al., 2003; Thompson et al., 1994). The phylogenetic tree was constructed using MEGA X to group the clones with following matrix: DNA weight matrix = IUB, transition weight = 0.50, delay divergent cutoff 30% (Tamura et al., 2007). The evolutionary history was inferred by using the maximum likelihood method and the Tamura–Nei model (Tamura et al., 2007). The evolutionary distances were computed using the maximum composite likelihood method and are in the units of the number of base substitutions per site. Evolutionary analyses were conducted in MEGA X using the Matrix Representation (Kumar et al., 2018) with Parsimony method (Ragan, 1992). The numbers on the nodes represent the bootstrap values as percentages from 1000 replicates.

Results:

The Table 2 presents the morphological characteristics of various *Ruellia* species identified in the study. Each row corresponds to a different species or subspecies,

Commented [MS14]: Please make separate headings for PCR amplification and sequencing; and Data analysis

Commented [MS15]: Please mention the city, and country name for all the equipment and consumables used in the study.

Formatted: Font: Italic

Formatted: Font: Italic

Commented [MS16]: City and country?

Commented [MS17]: Version please.

detailing their specific traits such as plant height, color, stem characteristics, leaf shape, pedicel height, calyx shape, corolla color, and fruit features.

Table 2. Morphological characteristics of plant species recorded during the study

Commented [MS18]: Make it a supplementary table

Taxa	Height Of the Plant M	The Plant Color	Stem					leaf shape	pedicel Height(cm	Calyx shape	Corolla color	Fruit		
			Internode shorter	Glabrous	Pubescent	Cylindrical	quadrangular					Petiole	Color before ripe	Shape
R.sp.Abha	23.3	green	+	+	-	-	+	lanceolate	2	lanceolate	Violat	+	Green	Elliptic, narrowing from below
R.sp.24651	3.7	green	-	-	+	+	+	Ovate	1	Elliptic	Violat&white	-	Green	oval with a narrow end
R.sp.24652	11.5	green	-	+	-	-	+	Linear	1	Linear	Rose	+	Green & Red	Cylindrical narrowing from above and below
R.simplex - R.malacosperma	8.3	Brown-purple	-	+	-	-	+	lanceolate	1.2	Linear	violat	+	Green & Red	Oblong
R.patula	5.5	green	-	-	+	-	+	Cordate	1	Linear	Violat&white	+	Green	Orbicular to ovule
R.sp.24650-rose	10.9	green	+	+	-	-	+	Linear	0.8	Elliptic	Pale rose	+	Green	Cylindrical, narrowing at the top
R.sp.24650-violat	12.8	Brown-purple	-	+	-	-	+	Linear	0.8	Elliptic	violat	+	Green	Fusiform
R.sp.24650-white	15.7	green	+	+	-	-	+	Linear	2cm	Elliptic		+	Green	elliptic narrowing from below

DNA Barcoding ~~Identification~~:

The table offers a detailed overview of the genetic ~~analysis~~ discrimination of various *Ruellia* species collected from different sites in the region. The analysis aims to accurately identify the species by matching ~~specific genetic~~DNA barcode markers. Four genetic markers were used: Psbk- psbi, Trnh-psba, Atpf-Atph, and RbcL. For each marker, the table lists the matched species and the corresponding accession number (**Table 3**). The collection from Abha identified by two primers, Psbk-psbi and AtpF-AtpH, identified the species as *R. brittoniana*, while TrnH-psbA identified it as *R. simplex* and Rbcl identified it as *R. tweediana*. Similarly, the sample *Ruellia* sp. 24651 was identified as *R. brittoniana* by Psbk-psbi and AtpF-AtpH, as *R. patula* by TrnH-psbA, and as *R. sp. sh2010* by Rbcl. Thesample *Ruellia sp. 24652* was consistently identified as *R. brittoniana* by three markers and as *R. carolinensis* by one marker. Similarly, *Ruellia malacosperma* was identified as *R. brittoniana* by three markers and as *R. sp. sh2010* by one marker. *Ruellia patula* was identified as *R. brittoniana* by three markers and as *R. patula* by one marker. *Ruellia sp. 24650 rose* was consistently identified as *R. brittoniana* by three markers and as R. tweediana by one marker. *Ruellia* sp. 24650 violet was identified as *R. brittoniana* by three markers and as *R. tweediana* by one marker. *Ruellia* sp. 24650 with was identifiedas *R. brittoniana* by one marker and as *R. tweediana* by two markers. The application of barcode markers estimates the genetic diversity and highlights potential identification issues within *Ruellia* species, demonstrating the importance of using multiple barcoding markers for accurate species identification.

The genetic analysis of *Ruellia* species collected from Abha and various other sites revealed diverse identifications based on different genetic markers. For the Abha collection, two primers (Psbk-psbi and AtpF-AtpH) identified the species as *R. brittoniana*, while TrnH-psbA identified it as *R. simplex* and Rbcl as *R. tweediana*.Similarly, *Ruellia sp*. 24651 was identified as *R. brittoniana* by Psbk-psbi and AtpF- AtpH, as *R. patula* by TrnH-psbA, and as *R. sp. sh2010* by Rbcl. Other samples, such as *Ruellia sp*. 24652, *Ruellia malacosperma*, *Ruellia patula*, and various *Ruellia sp. 24650* variants, showed consistent identifications by some markers and differing identifications by others. This highlights the genetic diversity and potential identification challenges

Commented [MS19]: Which Table??

Commented [MS20]: Please mention the region and country

Commented [MS21]: These are not the usual DNA/genetic markers, these are the barcode primers at conserved regions. Please mention as barcode markers.

Commented [MS22]: Please write the gene name properly ***trnH-psbA***

within *Ruellia* species, underscoring the importance of using multiple barcoding markers for accurate species identification.

The two markers (psbK-psbI & AtpF-AtpH) showed similar identification of the *Ruellia* sp, however, the other two primers TrnH-psbA & Rbcl) showed diverse results. For that purpose, wo combine the sequence of TrnH-psbA & Rbcl and construct the dendrogram to determine the similarity of the isolates (Table 4; Figure 10). The combined sequence analysis identified several *Ruellia* species, including *Ruellia* sp. *Abha* (*R. tweediana*), *Ruellia* sp. 24651 (R. sp. sh2010), *Ruellia* sp. 24652 (*R. carolinensis*), *Ruellia malacosperma* (*R. simplex*), *Ruellia patula* (*Ruellia patula*), *Ruellia* sp. *24650.rose* (*R. tweediana rose*), *R. tweediana* violet (*Ruellia sp. 24650 violet*), and *R. tweediana* white (*Ruellia sp. 24650 white*). The combination of two different marker regions provided more accurate information about the species, allowing for precise identification and classification.

Table 3. DNA barcoding results and their accession Numbers

Commented [MS23]: The results are not well presented. Please follow some DNA barcoding papers and interpret the results….Please see..https://doi.org/10.1038/s41598-023-40797-z

**Collection Site**		**Psbk-psbi**	**Trnh-psba**	**Atpf-Atph**	**RbcL**
Ruellia sp.Abha	Matched species	*R. brittoniana*	*R. simplex*	*R. brittoniana*	*R. tweediana*
	accession number	**OP893975**	**OP681139**	**OP893974**	**OP480015**
Ruellia sp.24651	Matched species	*R.brittoniana*	*R.patula*	*R.brittoniana*	*R.sp.sh2010*
	accession number	**OP503876**	**OQ095390**	**OP503877**	**OP867066**
Ruellia sp.24652	Matched species	*R.brittoniana*	*R.simplex*	*R.brittoniana*	*R.carolinensis*
	accession number	**OP745454**	**OP947575**	**OP745452**	**OP867067**
Ruellia malacosperma	Matched species	*R.brittoniana*	*R.simplex*	*R.brittoniana*	*R.sp.sh2010*
	accession number	**OP745458**	**OP745457**	**OP745456**	**OP745455**
Ruellia patula	Matched species	*R.brittoniana*	*R.patula*	*R.brittoniana*	*R.brittoniana*
	accession number	**OP480013**	**OQ106916**	**OP480014**	**OP745453**
Ruellia sp.24650.rose	Matched species	*R.brittoniana*	*R.simplex*	*R.brittoniana*	*R.tweediana*
	accession number	**OP745448**	**OP745449**	**OP745450**	**OP745451**
Ruellia sp.24650violat	Matched species	*R.brittoniana*	*R.simplex*	*R.brittoniana*	*R.tweediana*
	accession number	**OP712209**	**OP712210**	**OP712211**	**OP712208**
Ruellia sp.24650.withe	Matched species	*R.brittoniana*	R.tweediana	*R.brittoniana*	*R.tweediana*
	accession number	None	**OP712207**	**OP745447**	**OP712206**

Table 4. identification results after combining both molecular markers (Trnh-psba and rbcl)

Taxa	Taxa after combining)Trnh-psba and rbcl(
Ruellia sp.Abha	*R.tweediana*
Ruellia sp.24651	*R.sp.sh2010*
Ruellia sp.24652	*R.carolinensis*
Ruellia malacosperma	*R.simplex*
Ruellia patula	*R.patula*
Ruellia sp.24650.rose	*R.tweediana.rose*
Ruellia sp.24650.violat	*R.tweediana.violat*
Ruellia sp.24650.withe	*R.tweediana.white*

Discussion:

This study revealed that the herbaceous, perennial, and branching *Ruellia* species collected from the KSA are consistent with previous studies. The findings aligns with Migahid's (1974) study, Collenette (1999) type species and (Chaudhary, 2000) report on *Ruellia patula*. Similarly, Hupp et al. (2010), Chaudhary (2000), and Tripp (2010) reported similar observations on *Ruellia sororia*, which Kew Garden refers to as synonym with *Ruellia brittoniana* were corroborated. The study also indicates that stem and leaf hairiness ~~varies~~vary among species, with *R. patula* and *R. sp. 24651* having stems completely covered in hair.

Commented [MS24]: Then how you are claiming as the first report?

This study showed that the plants of *Ruellia* species collected from the KSA are herbaceous, perennial, and branching, consistent with the previous research. These findings align with Migahid, (1974), Collenette, (1999) and (Chaudhary (2000) on *Ruellia patula*, as well as Hupp et al., (2010), Chaudhary, (2000) and Tripp, (2010) on *Ruellia sororia*, which ~~Kew garden~~Kew Garden refers to as the synonym with *Ruellia brittoniana*.The study also found that stem and leaf hairiness ~~varies~~vary among different species, with stems of *R. patula* and *R. sp.24651* being completely covered in hairs. This result agreed with Santos, (2018) in his study of *Ruellia asperula.* Additionally, some species, such as *R. sp. Abha*, and *R. sp.24650* (rose-white) have prominent nodes. This result aligns with Hupp et al., (2010) study of the type *Ruellia tweediana*. The current study also showed that the leaves of all the examined species are simple, petiole and complete (Chaudhary,

2000). However, leaf shapes and edges vary among species. They range from lanceolate in *R. malacosperma* and *R. sp. Abha* (Long, 1966), to heart-to-ovate in *Ruellia patula*. Ram et al. (2015) also observed oval shapes in *R. sp.24651* and bar shape in *R. sp.24652* and *R. sp.24652.R. sp.24650* (rose-violet-white) consistent with this results. The presence, quantity, and distribution of hairs ~~vary~~ on the leaves ~~vary~~, with some species like *R. patula* and *R. sp. 24651* having entire leaves covered in hairs, while others like *R. malacosperma* have hairs concentrated on midrib and leaf margins. This observation agrees with Chaudhary (2000) findings in his study of *Ruellia patula*, *Ruellia malacosperma*, *Ruellia prostrata* and *Ruellia grandiflora*.

The plants collected in this study showed that the flowers in all types of *Raullia* are arranged on the stem in limited, two-branched inflorescences. They are five-parted, single, symmetrical, hermaphroditic, funnel-shaped, covered with glandular hairs. Color varied among species: pale pink in *R. malacosperma*, pale pink color in *R. sp.24652*, dark pink color in *R. sp.24650.rose*, white color in *R. sp.24650*.white and a mixture of white with violet color in *R. patula and R. sp.24651*. This finding corroborates Gilman's (1999) study on *Ruellia brittoniana*, Hupp et al.'s (2010) study on *Ruellia tweediana*, and Wilsonet al.'s (2020) study on *Ruellia simplex*. Additionally, stems and leaves of some species, such as *R. malacosperma* and *R. sp. 24650* (violet), turn purple when exposed to high temperatures, as observed by Hupp et al. (2010). All analyzed samples exhibited aconsistent cup with fruit and distinct hairy sepals~~,~~ featuring transparent membranous margins. Shapes varied, ranging from lanceolate in *R. sp. Abha* to threadlike in *R. patula* and *R. malacosperma - R. sp. 24652*, and ovoid in species *R. sp. 24650* (white, rose, violet) and *R. sp. 24651*. This finding aligns with Ezcurra's (1993) study on *R. brachysiphon* and *R. angustiflora* species, as well as with Chaudhary's (2000) and De Oliveira Chagas and da Costa-Lima's (2020) research on *Ruellia jiboia*.

The flowers have five fused petals, funnel-shaped, with different colors, which differ according to the type, and the reason may be due to the different colors of the corolla in response to the amount of sunlight and fertilizers they get in the soil. The resultof this study is consistent with the study of Wilson, et al., (2020) on *R. simplex*.

All the studied species contained four smooth stamens above a petal, fertile, of the long-two type. As for the pistil in all species, it consisted of an ovary comprising two lodgings,

and the ovules were in an axial placental position, elliptical in shape in all species except for *R.sp.24651*, where the ovary appeared. It is spherical to ovoid in shape, at the base of the ovary, there is a nectar disc, and the stigma is single, covered with hairs, and the stigma is in two parts and takes on a white to pale pink color.

The fruit is a simple dry open capsule, ranging from ovoid to spherical in *R. patula-R. sp. 24651*, and oblong-ovate in *R. sp. 24650* (rose-violet-white), *R. sp. 24652*, and *R. sp. Abha*, with an elongated oval shape in *R. malacosperma*. All fruits exhibit an upper prominence, initially green before ripening, and turning brown upon ripening. The seeds inside are equipped with hooks, which, upon exposure to moisture, cause the fruitto burst, dispersing the seeds (Chaudhary, 2000; Ezcurra, 1993; Barz et al., 2022; Tripp, 2007).

Johnson & Soltis, (1994) proposed molecular barcoding markers such as matK, rbcL, and psbA-trnH that exhibit a higher rate of nucleotide substitutions, making them one of the most rapidly evolving protein-coding regions of chloroplast DNA (cpDNA). However, molecular markers are evolving more rapidly than coding regions, with psbA-trnH being one of the fastest evolving markers in chloroplast DNA (cpDNA) (Sun, et al., 2013), and the most viable candidate for obtaining a single-site barcode for plants (Kress, et al., 2005). The perfect standard barcode sequence is still not found in plants so a combination of two or more plastid barcode barcodes is used to identify and distinguish species (Khan, et al., 2019), and it is worth noting that both *R.tweediana* and *R.simplex* species , *R.brittoniana* and *R.malacosperma* are only one species, according to Wunderlin et al., (2019); Hupp, et al., (2010).

The final result that we reached, after coding the DNA of all the studied plant samples, is that the eight species are only five species: *R.tweediana, R.sp.sh2010, R.carolinensis, R.simplex, R.patula,* and that the species collected from the region Taif, and it has flowers of different colors. It is only one species, and the difference in the color of the corolla may be due to it in response to environmental changes such as the photoperiod to which the plant was exposed or the soil pH, as mentioned by Wilson, et al., (2020).

Previous genetics studies have reported similar results, such as Kress & Erickson, (2007); McDade, et al., (2000); Potkar, Pardeshi, & Jadhav (2013); Khan, et al., (2019); Loera-Sánchez, et al., (2020); (Tripp, 2007). The former, while studying some species of the

Acanthaceae family, suggested that the rbcl barcode sequence is more effective, while a combination of matk, trnh-psba, can be used to study and determine the phylogeny of plants. The psbA-trnH trees proved more effective in resolving close species relationships within the genus *Ruellia* compared to other markers. This enhanced resolution might stem from these markers originating from the chloroplast genome and sharing a similar evolutionary history (Asmussen et al., 2006).

The latter recommended a combination of the psbA-trnH and rbcl cassettes as a universal barcode for interspecies discrimination, the trnH-psbA cassette will result in valid species counting where rbcL lacks discriminatory power, especially in species-rich genera of angiosperms. Both sites have standard primers currently available that make them universally amplifiable with minimal effort in the widest range of wild plants. This two-site plant barcode is now being applied to build a library of more than 700 species of the world's most important medicinal plants (Wiersema & León, 2007). The trnH-psbA and rbcl sequences were combined to accurately identify the samples. Combining the non-coding trnH-psbA sequence with one of three coding regions, rbcL, rpoB2 or rpoC1, offers the highest the highest comprehensiveness and greater ability to discriminate between species pairs. Complementing a rapidly evolving locus such as the trnH-psbA with a more conservative locus such as rbcL can reduce type 1 errors (Kress & Erickson, 2007). In this study, we followed this approach by combining both trnH-psbA and rbcL sequences.

Conclusion:

The species *Ruellia~~R.~~ sp. SH2010* was identified in the Asir region through DNA coding using the rbcL sequence, which requires further study to determine its exact classification. Several researchers have noted the absence of an ideal standard marker sequence for identification and recommend ~~using a combination of~~combining sequences to achieve more accurateresults. This study suggests that it is better to use a mixture of molecular markers, particularly trnH-psbA, to obtain the most precise identification possible.

Commented [MS25]: In conclusion, please mention the efficacy of the barcode genes on *Ruellia* species discrimination. Also, please establish a correlation between the morphological and barcode markers, which was missing throughout the MS.

Acknowledge:

The authors extend their appreciation to the Deanship of Research and Graduate Studies at King Khalid University for funding this work through small group research under grant number RGP1/200/45.

Reference

Commented [MS26]: Please cross check the references as per the journal standard.

Afzal, K., Uzair, M., Chaudhary, B. A., Ahmad, A., Afzal, S. A. M. I. N. A., and Saadullah, M. (2015). Genus Ruellia: Pharmacological and phytochemical importance in ethnopharmacology. Acta Poloniae Pharmaceutica-Drug Research, 72(5)PP.821

Alfarhan, A. H., Al-Turki, T. A., and Basahy, A. Y. (2005). Flora of Jizan region. Final Report Supported by King Abdulaziz City for Science and Technology, 1(2), 545.

Ali, M. A., Gyulai, G., Hidvegi, N., Kerti, B., Al Hemaid, F. M., Pandey, A. K., and Lee, J. (2014). The changing epitome of species identification–DNA barcoding. Saudi journal of biological sciences, 21(3), 204-231

Asmussen, c., Dransfield, J., Deickmann, V., Barfod, A., Pintaud, J., & Baker, W. (2006). A new subfamily classification of the palm family (Arecaceae): evidence from plastid DNA phylogeny. Botanical Journal of the linnean Society, pp. 15-38.

Braz, D. M., Freitas Azevedo, I. H., and Tripp, E. A. (2020). A New Species and Lectotypification in *Ruellia* (Acanthaceae) from the Southeastern Atlantic Forest, Brazil. Systematic Botany, pp. 334-339.

Chaudhary , S. (2000). Flora the Kingdom of Saudi Arabia Illustrated National herbarium ministry of agriculture and water Kingdom of Saudi Arabia. Riyadh. Saudi Arabia.

Chiu, N. Y., and Chang, K. H. (1995). The Illustrated Medicinal Plants of Taiwan, SMC Publishing Inc., Taipei, Taiwan , p.285

Collenette, S. (1999). Wildflowers of Saudi Arabia. National Commission for Wildlife Conservation and Development (NCWCD).

De Oliveira Chagas, E. C., and da Costa-Lima, J. L. (2020). Five new species of *Ruellia* (Acanthaceae) from the Atlantic Forest of northeastern Brazil. Neodiversity, pp. 39-55.

Ezcurra, C. . (1993). Systematics of *Ruellia* (Acanthaceae) in southern south America. Annals of the Missouri Botanical Garden 1990, pp. 787-845.

Gilman, E. F. (1999). *Ruellia* brittoniana. institut of food and agricultural sciences.

Graham, J. G., Quinn, M. L., Fabricant, D. S., and Farnsworth, N. R. (2000). Plants used against cancer–an extension of the work of Jonathan Hartwell. Journal of ethnopharmacology, pp. 347-377.

Gregory, T. R. (2005). DNA barcoding does not compete with taxonomy. Nature, 434(7037),1067-1067.

Hupp, K. V., Fox, A. M., Wilson, S. B., Barnett, E. L., and Stocker, R. K. (2010). Natural area weeds: Mexican petunia (*Ruellia* tweediana). EDIS, 2010(2).
Johnson, L., & Soltis, D. (1994). matK DNA sequences and phylogenetic reconstruction in Saxifragaceae s. str. Systematic botany, pp. 143-156
Khan, I., Shinwari, Z., Zahra, N., Jan, S., Shinwari, S., & Najeebullah, S. (2019). DNA barcoding and molecular systematics of selected species of family Acanthaceae. Pak. J. Bot, pp. 205-212.
Kress, w., & Erickson, D. (2007). A two-locus global DNA barcode for land plants: the coding rbcL gene complements the non-coding trnH-psbA spacer region.. PLoS one, p. 508.
Kress, W., Wurdack, K., Zimmer, E., Weigt, L., Janzen, D., (2005). Use of DNA barcodes to identify flowering plants. Proceedings of the National Academy of Sciences, pp. 8369-8374.
Thompson, J. D., Higgins, D. G., & Gibson, T. J. (1994). CLUSTAL W: improving the sensitivity of progressive multiple sequence alignment through sequence weighting, position-specific gap penalties and weight matrix choice. *Nucleic acids research*, *22*(22), 4673-4680.
Tamura, K., Dudley, J., Nei, M., & Kumar, S. (2007). MEGA4: molecular evolutionary genetics analysis (MEGA) software version 4.0. Molecular biology and evolution, 24(8), 1596-1599.

Kirtikar, K. R. B. B., and Basu, B. D. (1935). Indian medicinal plants. Indian Medicinal Plants.
Kumar, S., Stecher, G., Li, M., Knyaz, C., & Tamura, K. (2018). MEGA X: molecular evolutionary genetics analysis across computing platforms. *Molecular biology and evolution*, *35*(6), 1547.

Li, X., Yang, Y., Henry, R. J., Rossetto, M., Wang, Y., and Chen, S. (2015). Plant DNA barcoding: from gene to genome. Biological Reviews, 90(1), 157-166.
Loera-Sánchez, M., Studer, B., Kölliker, R., Kölliker, R, Kölliker, (2020). DNA barcode trnH-psbA is a promising candidate for efficient identification of forage legumes and grasses. BMC research notes, pp. 1-6.
McDade, L., Daniel, T., Masta , S., Riley, K., Daniel, (2000). Phylogenetic relationships within the tribe Justicieae (Acanthaceae): evidence from molecular sequences,morphology, and cytology. Ann. Missouri Botanical Garden, pp. 435-458.
Migahid AM (1974). Flora of Saudi Arabia. 1st edition King Saud University Press, Riyadh.
Potkar, V., Pardeshi , S., & Jadhav, P. (2013). Pattern of diversity using molecular marker (nr ITS): An example from Acanthaceae and Euphorbiceae. Int. J. Pharm. Res.Sch, pp. 1-4.
Ram, M., Rao, K., Alam, A., Singh, K. S., Ram, M., and Rao, K. (2015). Preliminary phytochemical analysis of different extracts of *Ruellia* patula, Luffa aegyptiaca and Thunbergia alata. Chem. and Pharm. Res, pp. 315-20.

Reddy, M. B., Reddy, K. R., and Reddy, M. N. (1991). Ethnobotany of Cuddapah district, Andhra Pradesh, India. International journal of pharmacognosy, pp. 273-280.

Samy, M. N., Khalil, H. E., Wanas, A. S., Kamel, M. S., Sugimoto, S., Matsunami, K., and Otsuka, H. (2013). Chemical constituents from the leaves of Ruellia tuberosa. Chemistry of Natural Compounds , pp.175-176.

Saitou N, Nei M (1987) The neighbor-joining method: a new method for reconstucting phylogenetic trees. Molecular Biological Evolution 4:406–425

Santos, M. T. (2018). Estudo farmacobotânico dos órgãos vegetativos de *Ruellia* asperula (Mart. and News) Lindas (ACANTHACEAE).

Selvam, A. (2008). Inventory of vegetable crude drug samples housed in botanical survey of India, Howrah. Pharmacognosy Reviews , pp. 61.

Sun, X., Bai, M., Yao, H., Guo, J., Li, M., & Hang, Y. (2013). DNA barcoding of populations of Fallopia multiflora, an indigenous herb in China. Genet Mol Res, pp. 4078-4089.

Suzuki, J. Y., Matsumoto, T. K., Keith, L. M., and Myers, R. Y. (2014). The chloroplast psbK-psbI intergenic region, a potential genetic marker for broad sectional relationships in Anthurium.journal Hortscience, 49(10),pp.1244-1252.

Thompson, J. D., Gibson, T. J., & Higgins, D. G. (2003). Multiple sequence alignment using ClustalW and ClustalX. *Current protocols in bioinformatics* (1), 2.3. 1-2.3. 22.

Tripp, E. A. (2007). Evolutionary relationships within the species-rich genus *Ruellia* (Acanthaceae). Systematic Botany, 32(3), 628-649.

Tripp, E. A. (2010). Taxonomic revision of *Ruellia* section Chiropterophila (Acanthaceae): a lineage of rare and endemic species from Mexico. Systematic Botany, pp. 629-661.

Tripp, E. A., Daniel, T. F., Fatimah, S., and McDade, L. A. (2013). Phylogenetic relationships within Ruellieae (Acanthaceae) and a revised classification. International Journal of Plant Sciences, 174(1), 97-137.

Wang, W., Wu, Y., Yan, Y., Ermakova, M., Kerstetter, R., and Messing, J. (2010). DNA barcoding of the Lemnaceae, a family of aquatic monocots. BMC Plant Biology, 10(1),pp. 1-11.

Wiersema , J., & León , B. (2007). World Economic Plants. USDA, ARS, National Genetic Resources Program. Germplasm Resources Information Network - (GRIN). Beltsville, Maryland: National Germplasm Resources Laboratory. usa: USDA, ARS, National Genetic Resources.

Wilson, s. b., Burkhead, a., Adams, c. R. , Freyre, R. , Freyre, and Freyre. (2020). Natural Area Weeds: Mexican Petunia (*Ruellia* simplex). UF/IFAS Extension,.

Wunderlin, R. P., Hansen, A. R., and Essig, F. B. (2019). Atlas of Florida Vascular Plants Institute for Systematic Botany. Univ of South Florida, Tampa.

Data Accessibility and Benefit -Sharing

Sequence reads and date are deposited to (NCBI)

National Center for Biotechnology Information (nih.gov).

All data have been shared with the broader public via appropriate biological databases.

Benefits from this research accrue from the sharing of our data and results on public databases as described above.

Figure Click here to access/download;Figure;Figures Tiff.docx

Figures(Tiff)

Table 1. List of Plant samples of the genus *Ruellia* studied and collection sites in Saudi Arabia

* Herbarium number

Taxa	Collection place	Collection date	GPS coordinates
1. *Ruellia sp.Abha*	Abha	December, 2021	18°16'37.6"N 42°43'23.5"E
2. *Ruellia sp.24651**	Aseer	December, 2021	19°07'44.5"N 41°55'42.2"E
3. *Ruellia sp.24652**	Jazan	September, 2021	16°59'30.7"N 42°42'59.0"E
4. *Ruellia malacosperma*	Riyadh, Jazan, Madinah	September, 2021	24°43'07.9"N 46°37'24.4"E
5. *Ruellia patula*	Jazan	September, 2021	16°54'50.1"N 42°33'20.4"E
6. *Ruellia .sp.24650rose**	Taif	December, 2021	21°30'34.8"N 40°29'17.2"E
7. *Ruellia.sp.24650.* Violet*	Taif	December, 2021	21°30'34.8"N 40°29'17.2"E
8. *Ruellia*.sp.24650 White	Taif	December, 2021	21°30'34.8"N 40°29'17.2"E

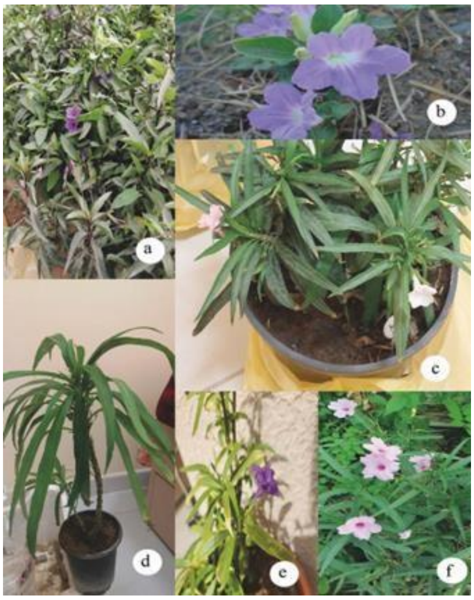

Figure 1: plants in their apparent shape

(a) *R. malacosperm* (b) *R. patula* and *R. sp.24651*. (c) *R. sp.24650 rose* and *R*.sp.24650 White (d) *R. sp Abha* (e) *R.sp.24650.* Violet

(f) *R. sp.24652*

Commented [MS27]: Please use high quality TIFF images

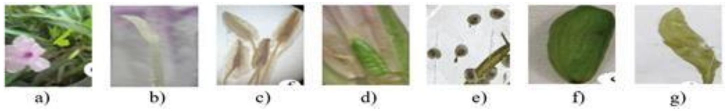

Figure 2. ***R.sp.Abha*** (a) flower shape, (b) stigma ,style (c) stamens (d)ovary (e) seeds (f) fruits (g) leaf

Commented [MS28]: Please make a picture panel of the Fig.2-9

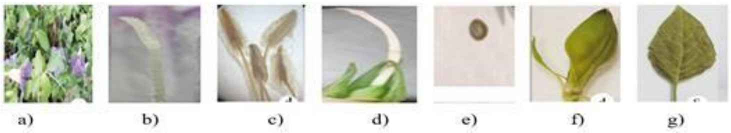

Figure 3. ***R.sp.24651*** (a) flower shape, (b) stigma, style (c) stamens (d) ovary (e) seeds (f) fruits (g) leaf

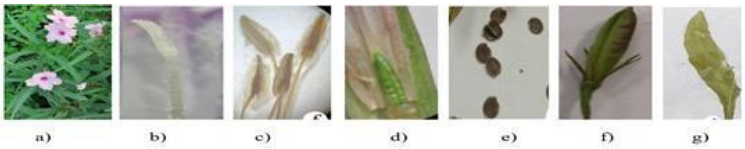

Figure 4. ***R.sp.24652- R. carolinensis*** (a) flower shape (b) stigma, style (c) stamens (d)ovary (e) seeds (f) fruits (g) leaf

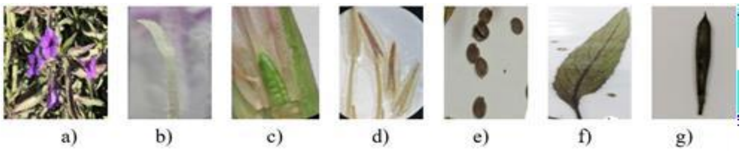

Figure 5 ***R.simplex - R.malacosperma*** (a) flower shape, (b) stigma, style (c) stamens (d) ovary (e) seeds (f) fruits (g) leaf

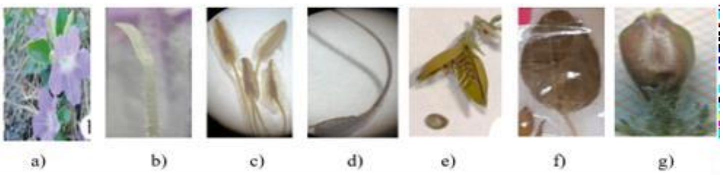

Figure ***6 R. patula*** (a) flower shape (b) stigma, style (c) stamens (d) ovary (e) seeds (f) fruits (g) leaf

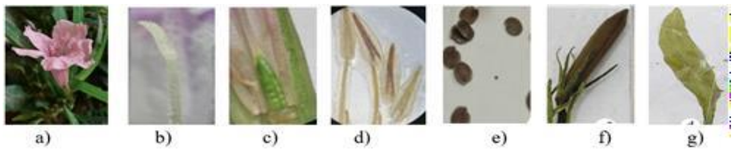

Figure 7 ***R.sp.24650-rose*** (a) flower shape, (b) stigma, style (c) stamens (d) ovary (e) seeds (f) fruits (g) leaf

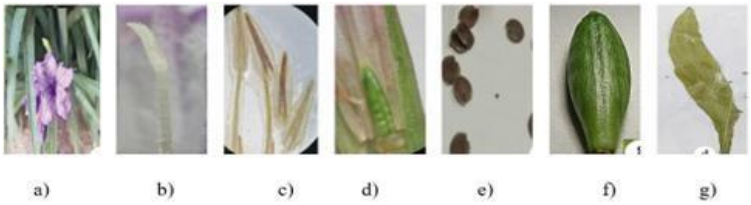

Figure 8 ***R.sp.24650-violat*** (a) flower shape, (b) stigma ,style (c) stamens (d)ovary (e) seeds (f) fruits (g) leaf

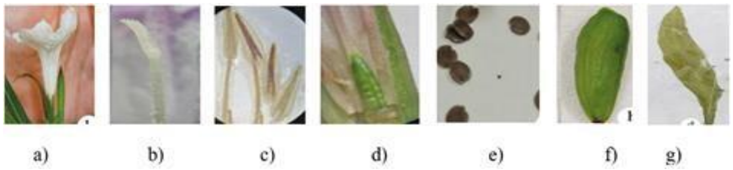

Figure **9** ***R.sp.24650-white*** (a) flower shape, (b) stigma, style (c) stamens (d) ovary (e) seeds (f) fruits (g) leaf

Table 2. Morphological characteristics of plant species recorded during the study

Taxa	Height Of the Plant M	The Plant Color	Stem					leaf shape	pedicel Height(cm	Calyx shape	Corolla color	Fruit		
			Internode shorter	Glabrous	Pubescent	Cylindrical						Petiole	Color before ripe	Shape
R.sp.Abha	23.3	green	+	+	-	-	+	lanceolate	2	lanceolate	Violat	+	Green	Elliptic, narrowing from below
R.sp.24651	3.7	green	-	-	+	+	+	Ovate	1	Elliptic	Violat&white	-	Green	oval with a narrow end
R.sp.24652	11.5	green	-	+	-	-	+	Linear	1	Linear	Rose	+	Green & Red	Cylindrical narrowing from above and below
R.simplex - R.malacosperma	8.3	Brown-purple	-	+	-	-	+	lanceolate	1.2	Linear	violat	+	Green & Red	Oblong
R.patula	5.5	green	-	-	+	-	+	Cordate	1	Linear	Violat&white	+	Green	Orbicular to ovule
R.sp.24650-rose	10.9	green	+	+	-	-	+	Linear	0.8	Elliptic	Pale rose	+	Green	Cylindrical, narrowing at the top
R.sp.24650-violat	12.8	Brown-purple	-	+	-	-	+	Linear	0.8	Elliptic	violat	+	Green	Fusiform
R.sp.24650-white	15.7	green	+	+	-	-	+	Linear	2cm	Elliptic		+	Green	elliptic narrowing from below

Commented [MS29]: Make it a supplementary table

Table 3. DNA barcoding results and their accession Numbers

Commented [MS30]: The tabular representation is not convincing… Please delete 'Matched species' and Accession number'...mention the species and acc. No in the parentheses.

Collection Site		Psbk-psbi	Trnh-psba	Atpf-Atph	RbcL
Ruellia sp.Abha	Matched species	*R. brittoniana*	*R. simplex*	*R. brittoniana*	*R. tweediana*
	accession number	**OP893975**	**OP681139**	**OP893974**	**OP480015**
Ruellia sp.24651	Matched species	*R.brittoniana*	*R.patula*	*R.brittoniana*	*R.sp.sh2010*
	accession number	**OP503876**	**OQ095390**	**OP503877**	**OP867066**
Ruellia sp.24652	Matched species	*R.brittoniana*	*R.simplex*	*R.brittoniana*	*R.carolinensis*
	accession number	**OP745454**	**OP947575**	**OP745452**	**OP867067**
Ruellia malacosperma	Matched species	*R.brittoniana*	*R.simplex*	*R.brittoniana*	*R.sp.sh2010*
	accession number	**OP745458**	**OP745457**	**OP745456**	**OP745455**
Ruellia patula	Matched species	*R.brittoniana*	*R.patula*	*R.brittoniana*	*R.brittoniana*
	accession number	**OP480013**	**OQ106916**	**OP480014**	**OP745453**
Ruellia sp.24650.rose	Matched species	*R.brittoniana*	*R.simplex*	*R.brittoniana*	*R.tweediana*
	accession number	**OP745448**	**OP745449**	**OP745450**	**OP745451**
Ruellia sp.24650violat	Matched species	*R.brittoniana*	*R.simplex*	*R.brittoniana*	*R.tweediana*
	accession number	**OP712209**	**OP712210**	**OP712211**	**OP712208**
Ruellia	Matched	*R.brittoniana*	R.tweediana	*R.brittoniana*	*R.tweediana*

sp.24650.withe	species				
	accession number	None	**OP712207**	**OP745447**	**OP712206**

Table 4. identification results after combining both molecular markers (Trnh-psba and rbcl)

Commented [MS31]: Elaborate the combination in the methods, results and discussion. Also, introduce the advantage of combination of two barcode primers in the introduction section.

**Taxa**	**Taxa after combining (Trnh-psba and rbcl)**
Ruellia sp.Abha	*R.tweediana*
Ruellia sp.24651	*R.sp.sh2010*
Ruellia sp.24652	*R.carolinensis*
Ruellia malacosperma	*R.simplex*
Ruellia patula	*R.patula*
Ruellia sp.24650.rose	*R.tweediana.rose*
Ruellia sp.24650.violat	*R.tweediana.violat*
Ruellia sp.24650.withe	*R.tweediana.white*

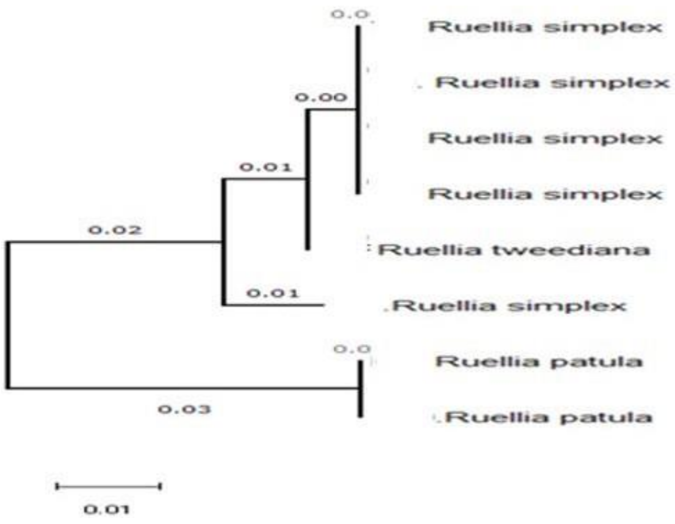

Figure 10. Phylogenetic tree constructed based on concatenated *Ruellia sp* .sequences along with reference sequence from NCBI using maximum likelihood and neighbor-joining with 1000 bootstrap value with Maga X.

Commented [MS32]: Please use high quality TIFF images

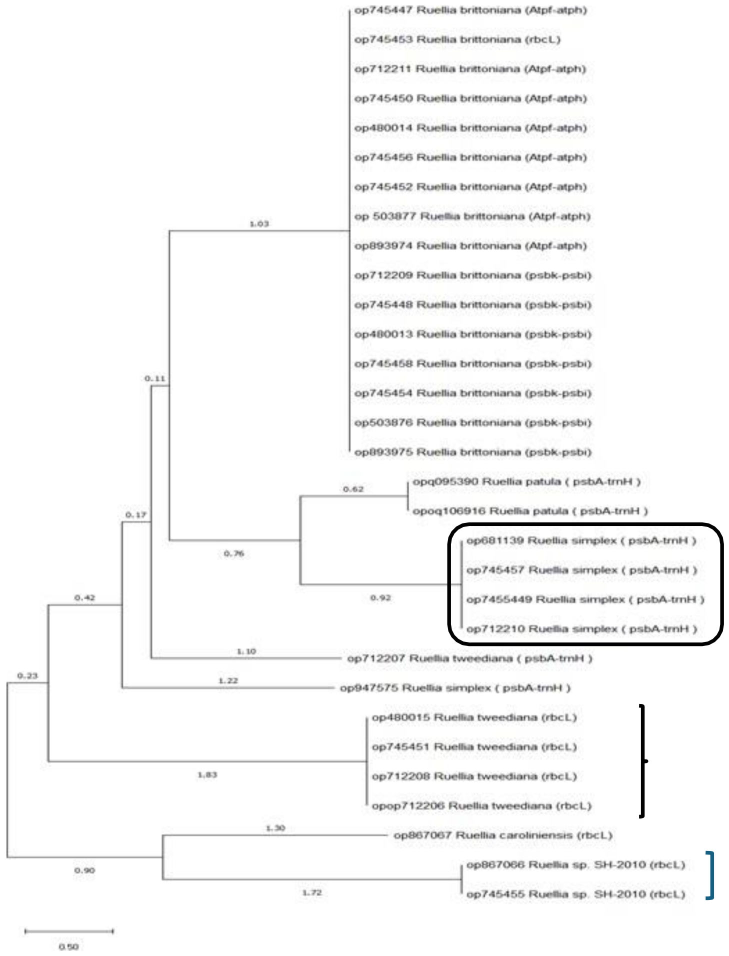

.

Figure11: Combine phylogenetic tree constructed using Maga X with 1000 bootstrap value of concatenated Psbk-psbi , Trnh-psba , Atpf-Atph and rbcL generated sequences from *Ruellia sp* and their represented sequences from NCBI. The percentage of bootstrap value from 1000 replicates is represented by the number of nodes. The horizontal bar represents the genetic distance

Commented [MS33]: Please use high quality TIFF images
