# Supplementary material for: Morphological characterization and DNA barcoding of Ruellia sp. in Saudi Arabia
Source: PLoS One. 2025 Feb 19;20(2):e0315827. doi: 10.1371/journal.pone.0315827 (PMC11838864; doi:10.1371/journal.pone.0315827)
Supplement: Attachment — Submitted filename: Response to Reviewers..docx [file pone.0315827.s004.docx]

**Response file for revised submission**

Thanks for providing us the opportunity to revise the manuscript “**Morphological characterization and DNA barcoding of *Ruellia sp* in Saudi Arabia**” in Plos-one. We welcome the comprehensive suggestions and positive recommendations. We try to reply to each point for the improvement of our manuscript based on the reviewers' feedback.

| **Reviewer 1** |  |
| --- | --- |
| **Comments** | **Response** |
| The manuscript (Morphological characterization and (DNA) Barcoding identification of *Ruellia* spp. (Acanthaceae) in Saudi Arabia) is clear, unambiguous, and technically correct. The paper contains interesting results and is generally well-written and structured. The experiments were successful, and the data was well understood and modeled in detail. | Thank you for the positive feedback on the manuscript titled "Morphological Characterization and (DNA) Barcoding Identification of Ruellia spp. (Acanthaceae) in Saudi Arabia." We are pleased to know that you found the work clear, technically correct, and well-structured, as well as the results interesting. |
| The introduction is appropriate, but a few things need further improvements, especially the study hypothesis that should be added for the last five years | Thank you for your valuable feedback. We appreciate your suggestion to improve the introduction. We revised the introduction section and hypothesis section and update the information. |
| The phrase "recognized as the first taxonomic investigation of *Ruellia* species within the Kingdom of Saudi Arabia" is somewhat repetitive, as "the first taxonomic investigation" already implies this recognition. | Thank you for your insightful comment. We agree that the phrase is indeed redundant. We will revise the sentence to remove the unnecessary repetition, simplifying it to "the first taxonomic investigation of Ruellia species within the Kingdom of Saudi Arabia" for clarity and conciseness. We appreciate your attention to detail in helping improve the manuscript. |
| The enumeration of species and regions (e.g., "Abha, Aseer (24651)...") is cluttered. Consider rephrasing for clarity, perhaps using bullet points or breaking into more precise sentences | This is a definition for unknown species due to they do not have a scientific name. e.g., “Abha, Aseer (24651)”, Abha, Aseer refers to the collection location of the sample, and the number (24651) refers to the deposit number of the sample in the King Saud University Herbarium. |
| The abstract does not provide a good outline for the study. | Thanks for the positive comment. We revised the title as per suggestion |
| What is the limitation of this study | A potential limitation of the study on the "Morphological Characterization and DNA Barcoding of Ruellia spp. in Saudi Arabia" could be related to incomplete sampling across diverse regions, limiting the representation of species. The efficacy of DNA barcoding might also be constrained by insufficient variability in the chosen barcode regions, complicating species differentiation, especially among closely related taxa. Additionally, morphological variability due to environmental factors and potential hybridization could obscure species identification. The absence of comprehensive reference sequences in global DNA databases may further hinder species confirmation. |
| Arrange the keywords in alphabetical order. | Thanks for the comment. We write the keywords in alphabetical order. |
| Cross-check the references in the text and reference cite | We cross check the references in the text and bibliography. |
| Ensure that the citation style remains consistent throughout the document. In the first section, "Migahid's (1974) study" differs from "(Migahid, 1974)" used later. | We cross check the citation style through out the MS. |
| Some sentences are pretty long and complex, which can be confusing. Breaking them into shorter sentences can improve comprehension. Additionally, ensuring consistent punctuation can aid in reader understanding. | Thanks for your positive feedback. We revised the long sentence into shorter with intended meaning. |
| Conclusion: Improve this part concerning formulated objectives | Thanks for your positive feedback and comment. We revised the conclusion aligned with formulated objectives. |
| **Reviewer #2** | |
| Comments | Response |
| The manuscript ‘Morphological characterization and (DNA) Barcoding identification of Ruellia spp. (Acanthaceae) in Saudi Arabia’ [PONE-D-24-30210] focused on the genetic discrimination of Ruellia spp. Using DNA barcoding. I have some suggestions for improvement of the manuscript which is cited in the track changed MS. The English language, grammar, and punctuation need to be cross-checked by a native English speaker. References need to be thoroughly checked. Figures may be reduced to 4-5 with high-quality TIFF Images. | The article revised for the for English editing, grammar check.  Reference was cross checked in the text and bibliography. |
| **Reviewer # 3** | |
| Comments | Response |
| Title:  The title of the paper is informative and relevant it is reflect the MS contents. | Thanks for your positive feedback for the MS |
| Abstract:  Sufficiently informative, covering all sections of the MS except methodology and clearly summarize the background, the aim of the study, results and conclusion. It reflects the article content. So, authors should supply a short statement describing the methods that was followed in the MS. | Thank you for your helpful feedback. We appreciate your acknowledgment of the summary's clarity in covering most sections of the manuscript. In response to your suggestion, we will include a concise statement in the abstract outlining the key methodologies used in the study. |
| It describes what the author hoped to achieve accurately; the research question is outlined and justified and clearly state the problem being investigated. It summarizes relevant research to provide context, and explain what other authors findings. | Thank you for your positive feedback. Your comments are encouraging, and we are pleased the manuscript meets these expectations. |
| Author/s gave what is already known about the use of DNA barcoding as an effective tool to identify diverse *Ruellia* species across various habitats | Thank you for your feedback. We are pleased that the manuscript effectively conveys the current knowledge on the use of DNA barcoding as an effective tool for identifying diverse Ruellia species across different habitats. We will continue to ensure that the discussion highlights the significance of this tool for species identification within the broader framework of plant taxonomy. |
| Methodology:  The description of materials and methods insufficiently informative to allow replication of the experiment. The statistical methods not mentioned in the MS. | We revised the materials & methods section and add the statistical methods. In addition, attach the Numerical analysis file. |
| Results:  The experimental results are insufficient to justify the conclusions. Results poor presented. | We revised the result and conclusion section. |
| Tables and figures  Tables not well presented  Figures lack scale bars | We revised the tables presentation.  We already have a scale bar for figures 3, 4 and 5 |
| Discussion and conclusions:  The discussion and conclusion are not supported by the results. | We revised the discussion and aligned with results and conclusion section |
| References update | Updated with references published 2022 |
| Comment 8 in pdf file | First: Samples No. (4-5) are previously known samples mentioned in classification books. To be sure, the morphological description of the samples matched what was mentioned in books such as the book of Chaudhry and Sheela, both of which mentioned them in a brief description. There was no need to put a number for them, as it is customary to put unknown samples to prepare for their identification. This is what was followed.  Second: As for sample No. (1), it is a rare sample, and during the collection, we were only able to obtain one sample, and it was used in the precise laboratory examination of its parts. We could not include it in the herbarium with a special number, but rather it was satisfied with its name, which we gave it by its location, the Abha region. |
| Comment 24 in pdf file | The study is the first taxonomic and molecular study at the level of the genus *Ruellia* in Saudi Arabia.  As for the similarity of morphology, the separator is the study of DNA or identification through it, and this is what we followed |
| Comment [ MS29] in pdf file | We are separated it in supplementary file, and captioned with Table S1: Morphological characteristics of plant species recorded during the study |
| **Journal Requirements** | |
| Acknowledgement: The authors extend their appreciation to the Deanship of Research and Graduate Studies at King Khalid University for funding this work through small group research under grant number RGP1/200/45. | We would like to include the acknowledgements at the end of the article as they are included in the published research of your journal. Such as (https://doi.org/10.1038/s41598-023-40797-z) |
| References | References were included using the EndNote program, but there is a problem that we were not able to include old references in any way except by adding them manually independently. |
| Use PACE | We tried to use this program several times to insert images; unfortunately we were unable to upload the images to the program. |
| Table and figures in the template | The figures and table are included as shown in the template and published papers. |
| Financial Disclosure | The Deanship of Research and Graduate Studies at King Khalid University has provided financial funding under grant number RGP1/200/45. The author(s) have not received this financial funding for this study yet now, and we will receive it after the publication is completed. |
| Author contributions | Supervision: MSA  Visualization: AEM  Designed, performed the experiments and writing: JSA and MA.  Analyzed the data: JSA.  Review and editing: MA, MAA, MDA |
